# Prognostic Value of the Platelet-to-Lymphocyte Ratio in Patients With Acute Coronary Syndrome and Its Correlation With Angiographic Findings

**DOI:** 10.7759/cureus.105558

**Published:** 2026-03-20

**Authors:** Deepak Bhat Seetharama, Kavya Prasad, Varsha Rakshitha Prakash, Megha Reddy, Mohamed Omar Shariff, Varun Vinayak Prakash Rao, Vadagenalli Sathyanarayana Rao Prakash, Sannidhi Nagesh Babu Donty, Meghana Anandamayi Rupanagunta

**Affiliations:** 1 Internal Medicine, M.S. Ramaiah Medical College, Bengaluru, IND; 2 Cardiology, M.S. Ramaiah Medical College, Bengaluru, IND

**Keywords:** acute coronary syndrome, biomarker, coronary angiography, major adverse cardiovascular event, neutrophil-to-lymphocyte ratio, platelet-to-lymphocyte ratio, syntax score

## Abstract

Introduction

Acute coronary syndrome (ACS) is a major manifestation of coronary heart disease and is associated with significant morbidity and mortality. Inflammatory and thrombotic mechanisms play an important role in its pathogenesis. The platelet-to-lymphocyte ratio (PLR) has emerged as a simple and readily available inflammatory marker that may have prognostic value. The present study aimed to evaluate the association between PLR and major adverse cardiovascular events (MACE) in patients with ACS.

Materials and methods

This prospective observational study was conducted at a tertiary care center after institutional ethics approval. A total of 640 consecutive patients aged more than 18 years presenting with ACS and meeting the inclusion and exclusion criteria were enrolled after obtaining informed consent. PLR was measured from blood samples collected within 12 hours of symptom onset and prior to reperfusion or heparin therapy. Demographic details, clinical history, and laboratory parameters were recorded. SYNTAX scores, PLR, and neutrophil-to-lymphocyte ratio (NLR) were calculated. MACE were defined as the occurrence of heart failure, recurrent myocardial infarction, or death during the in-hospital period. Patients were followed until discharge or in-hospital death. Patients with incomplete laboratory or outcome data were excluded from the final analysis. Multivariate regression analysis was performed to adjust for potential confounding variables.

Results

The mean age of the study population was 62.01 ± 11.66 years, with a female preponderance (59.22%). MACE occurred in 316 patients (49.38%). Patients with MACE had significantly higher PLR and NLR values and lower ejection fraction (EF) compared with those without MACE (p < 0.001). A higher proportion of patients with a SYNTAX II score greater than 22 experienced MACE compared with those with lower scores (80.20% vs 21.66%, p < 0.001). PLR demonstrated a significant positive correlation with NLR and SYNTAX I score and a negative correlation with EF. Similar associations were observed across ACS subgroups, including non-ST-elevation myocardial infarction, ST-elevation myocardial infarction, and unstable angina.

Conclusions

Higher PLR levels were associated with greater angiographic disease severity and an increased incidence of in-hospital MACE in patients with ACS. These findings suggest that PLR may serve as a useful adjunctive marker for risk assessment in ACS. Further multicenter prospective studies with longer follow-up are required to validate its prognostic role.

## Introduction

Globally, coronary heart disease (CHD) is a leading cause of morbidity and mortality. Cardiovascular diseases remain the leading cause of death worldwide. The World Health Organization estimates that 19.8 million people died from cardiovascular diseases in 2022, accounting for approximately 32% of all global deaths, with the majority attributable to heart attack and stroke [1]. Acute coronary syndrome (ACS) is a life-threatening manifestation of CHD, mainly caused by rupture of the coronary plaque and thrombus formation, leading to occlusion of the coronary artery. ACS includes a spectrum of conditions, including non-ST-elevation myocardial infarction (NSTEMI), ST-elevation myocardial infarction (STEMI), and unstable angina (UA). Approximately seven million people are diagnosed with ACS worldwide every year, with a reported in-hospital mortality rate of 5% [2]. The cited incidence and mortality figures in this paragraph refer to global data.

The unstable plaque in ACS is the main cause of death and leads to other major adverse cardiovascular events (MACE), such as reinfarction and recurrent ischemia [3,4]. In the present study, MACE was defined as the occurrence of heart failure (HF), recurrent myocardial infarction, or death during hospitalization. These events significantly increase morbidity and place a strain on scarce healthcare resources. Therefore, there has been a search for biomarkers and indices with prognostic efficacy for disease progression. This is crucial for identifying patients who require aggressive therapy and close monitoring. Various scoring systems have been developed, such as the SYNTAX I and II scores; however, these are composite scores of different parameters, which are time-consuming and require tedious calculations.

The relationship between inflammation and thrombosis is well known and critical in ACS [5]. Platelets play a key role in the pathogenesis of ACS. The platelet-to-lymphocyte ratio (PLR) is a novel inflammatory marker that also reflects thrombotic processes. It is an inexpensive, readily available indicator. Several inflammatory markers have been evaluated for risk stratification in ACS, including CRP and the neutrophil-to-lymphocyte ratio (NLR). However, these primarily reflect inflammatory burden and stress response. PLR is attractive because it additionally incorporates the platelet component, which is central to ACS pathogenesis through thrombosis and platelet activation. Furthermore, PLR is derived from routine complete blood counts, enabling rapid, low-cost implementation without additional testing. It is important to note, however, that elevated PLR is not specific to ACS and may also be observed in conditions such as infections, malignancies, or other chronic inflammatory states. Therefore, interpretation of PLR as a prognostic marker should always be made in the appropriate clinical context, with careful exclusion of potential confounding conditions.

Although prior studies have reported associations between PLR and adverse outcomes in ACS, there remains limited prospective evidence from South Asian cohorts, and relatively few studies have simultaneously evaluated PLR alongside objective angiographic severity measures such as the SYNTAX scoring system. In this context, data clarifying whether PLR reflects both anatomical disease burden and short-term in-hospital events could strengthen its potential role as an accessible adjunct for early risk stratification. The objective of the present study was to evaluate the association of PLR with angiographic severity (SYNTAX scores) and in-hospital MACE among patients presenting with ACS.

## Materials and methods

Study design and setting

This prospective, observational study was conducted under the Department of Cardiology, Ramaiah Medical College Hospital, Bangalore. Institutional Ethics Committee approval was obtained (MSRMC/EC/SP-01/05-2024). The study was conducted from May 2024 to October 2024. A total of 640 patients, aged more than 18 years of either gender, were included in the study after obtaining voluntary, written, informed consent. A sample size calculation was performed, and the final sample included all eligible patients recruited during the predefined study period.

Patients with a history of hematological disorders, recent blood transfusions, recent infections, chronic liver or kidney disease, on chemotherapy or anticoagulants, or with autoimmune diseases were excluded from the study. Pregnant patients and patients refusing to provide consent were also excluded. Recent infection was defined as any clinically documented infection occurring within the preceding four weeks. The term “anticoagulants” referred to therapeutic anticoagulation, such as warfarin or direct oral anticoagulants prescribed for conditions like atrial fibrillation or venous thromboembolism, and did not include standard antiplatelet therapy used in the management of ACS.

A convenience sampling method was adopted to select the subjects. Since the sampling frame of the population was not available, a nonrandom sampling method was used. Subjects visiting the inpatient or outpatient department of the tertiary care hospital within the four-month study period were included consecutively. Patients with incomplete laboratory or outcome data were excluded from the final analysis. Complete case analysis was performed, and no imputation techniques were applied.

Patient evaluation

Demographic details, relevant past history, and personal history were recorded. Patients were categorized as having STEMI, NSTEMI, or UA based on symptoms, cardiac biomarkers, and ECG findings in accordance with standard guidelines [6]. STEMI was defined as angina symptoms (e.g., chest pain radiating to the neck, jaw, or shoulders, with dyspnea, nausea/vomiting, or syncope) with elevated cardiac biomarkers and ST elevation on ECG. NSTEMI was defined as angina symptoms with elevated cardiac biomarkers and ECG abnormalities, but without ST elevation. UA was defined as angina symptoms without elevated cardiac biomarkers, or ischemic chest pain or equivalent symptoms consistent with ACS, without elevation of cardiac biomarkers and without new, persistent ST-segment elevation on the electrocardiogram.

Laboratory parameters

Blood samples were collected prior to reperfusion or heparin therapy from the median cubital, cephalic, or dorsal hand veins. Laboratory investigations were performed promptly upon the patient’s arrival at the hospital and within 12 hours of symptom onset. Complete blood counts, including platelet and differential counts, were measured using an automated hematology analyzer manufactured by Randox (Crumlin, UK). Internal quality control was performed using three levels of control samples twice daily, at approximately 8 AM and 8 PM, maintaining a 12-hour interval between runs to ensure the accuracy and reliability of measurements. Investigators assessing PLR were single-blinded. The proportion of lymphocytes in the differential count relative to the total WBC count was used to calculate absolute lymphocyte counts. PLR was calculated as platelet count ÷ lymphocyte count. NLR was calculated as neutrophil count ÷ lymphocyte count.

Coronary artery disease assessment

All patients underwent coronary angiography (CAG). Anatomic severity was assessed using SYNTAX I and SYNTAX II scores [7,8]. The SYNTAX II score was computed using the online calculator [9]. Angiograms were assessed by a single observer; interobserver variability was therefore not measured.

Follow-up and outcomes

Patients were followed until hospital discharge. MACE were defined as the occurrence of HF, recurrent myocardial infarction, or death during the in-hospital period. The relationship between PLR and NLR with the severity of coronary artery disease was analyzed across the three forms of ACS. No follow-up beyond the period of hospitalization was performed. Recurrent myocardial infarction was considered distinct from the index ACS event.

HF was considered a component of MACE when any of the following criteria were met during the index hospitalization: (1) clinical diagnosis of HF requiring hospitalization: new-onset or worsening HF leading to unplanned hospital admission, or intensification of HF therapy, such as initiation of intravenous diuretics, vasodilators, or inotropes; (2) clinical signs and symptoms consistent with HF: symptoms including dyspnea at rest or on exertion, orthopnea, paroxysmal nocturnal dyspnea, or fatigue, and signs including pulmonary rales, elevated jugular venous pressure, peripheral edema, or S3 gallop, with diagnosis supported by documentation from the treating physician; (3) objective evidence of cardiac dysfunction (at least one): echocardiographic findings such as reduced left ventricular ejection fraction (EF; <40%) or preserved EF with structural heart disease or diastolic dysfunction, or radiographic evidence of pulmonary congestion or interstitial edema; (4) biomarker support (when available): elevated natriuretic peptide levels (B-type natriuretic peptide or N-terminal pro-B-type natriuretic peptide) interpreted in the appropriate clinical context; and (5) standardized diagnostic framework: diagnosis aligned with established European Society of Cardiology/American College of Cardiology/American Heart Association guidelines or Framingham criteria where applicable.

Statistical analysis

Data were analyzed using IBM SPSS Statistics for Windows, Version 28.0 (Released 2021; IBM Corp., Armonk, NY, USA). The chi-square test was used for analyzing categorical data, while Student’s t-test was used for numerical data. Pearson’s coefficient was used to assess the strength of correlation. A p-value of less than 0.05 was considered statistically significant. Normality was assessed using graphical methods and statistical tests such as the Shapiro-Wilk or Kolmogorov-Smirnov test. As several continuous variables did not meet normality assumptions, the Mann-Whitney U test was used to compare two independent groups, and Spearman correlation analysis was used to assess relationships between continuous variables. Multivariate linear regression was performed to adjust for potential confounding variables. The regression coefficients with 95% CIs are reported in the results, both before and after adjusting for confounders. Unadjusted confounders were analyzed using simple linear regression.

## Results

The mean age of the study population was 62.01 ± 11.66 years, with a female preponderance (379 patients, 59.22%). Subgroup analyses were exploratory and should be interpreted with caution due to the increased chance of false-positive findings. The baseline characteristics are presented in Table 1.

**Table 1:** Demographic and baseline characteristics of the study population The age was presented as mean ± SD. Other demographic and baseline characteristics were presented as numbers and percentages. DM, diabetes mellitus; EF, ejection fraction; IHD, ischemic heart disease; MACE, major adverse cardiovascular events; NLR, neutrophil-to-lymphocyte ratio; NSTEMI, non-ST-elevation myocardial infarction; PLR, platelet-to-lymphocyte ratio; STEMI, ST-elevation myocardial infarction; U/A ratio, uric acid-to-albumin ratio; UA, unstable angina

Characteristic	N (%)/mean ± SD
Demographics	
Age (years)	62.01 ± 11.66
Female	379 (59.22%)
Comorbidities	
Hypertension	390 (60.94%)
Type 2 DM	354 (55.31%)
IHD	97 (15.16%)
Indication	
NSTEMI	219 (34.22%)
STEMI	215 (33.59%)
UA	206 (32.19%)
Outcome	
MACE present	316 (49.38%)
SYNTAX I score	13.28 ± 10.97
SYNTAX II score	21.52 ± 10.48
Total leukocyte count (/µL)	10,286.11 ± 3,783.88
Neutrophil count (/µL)	6,999.57 ± 3,435.54
Serum creatinine (mg/dL)	0.93 ± 0.44
Uric acid (mg/dL)	5.39 ± 1.70
Albumin (g/dL)	3.30 ± 0.90
U/A ratio	1.78 ± 0.84
Platelet count (/µL)	291,836.15 ± 85,791.50
Lymphocyte count (/µL)	2,445.76 ± 1,551.07
PLR	218.66 ± 113.81
NLR	3.70 ± 3.19
EF (%)	47.07 ± 8.19

When compared according to MACE, there was no significant difference in age or gender (p = 0.422 and 0.764, respectively). The uric acid-to-albumin ratio (U/A ratio), PLR, and NLR were significantly higher in patients with MACE than in those without MACE, while EF was significantly lower in patients with MACE (p < 0.001) (Table 2).

**Table 2:** Distribution of the laboratory findings and echocardiographic parameters according to MACE The asterisk (*) indicates a statistically significant p-value. The data were presented as mean ± SD. The unpaired t-test was used to assess statistical significance, and a p-value of less than 0.05 was considered statistically significant. EF, ejection fraction; MACE, major adverse cardiovascular events; NLR, neutrophil-to-lymphocyte ratio; PLR, platelet-to-lymphocyte ratio; U/A ratio, uric acid-to-albumin ratio

Parameter	MACE absent (mean ± SD)	MACE present (mean ± SD)	t-score	p-value
U/A ratio	1.89 ± 0.98	4.62 ± 0.66	-41.65	<0.001*
PLR	257.14 ± 125.51	782.24 ± 147.77	-48.40	<0.001*
NLR	12.45 ± 6.38	27.40 ± 2.48	-39.27	<0.001*
EF (%)	47.67 ± 7.60	26.46 ± 4.41	43.31	<0.001*

The duration of hospital stay was significantly longer in patients with MACE (8.40 ± 1.74 days) than in patients without MACE (2.77 ± 1.35 days; p < 0.001). SYNTAX scores were compared, and it was noted that the SYNTAX I score was significantly higher in patients with MACE (43.90 ± 8.07) compared with patients without MACE (15.18 ± 7.37; p < 0.001). The proportion of patients experiencing MACE among those with a SYNTAX II score greater than 22 was significantly higher than among those with a SYNTAX II score less than 22 (p < 0.002) (Table 3).

**Table 3:** Distribution of SYNTAX II score according to MACE The asterisk (*) indicates a statistically significant p-value. The data were presented as numbers and percentages. The chi-square test was used to assess statistical significance, and a p-value of less than 0.05 was considered statistically significant. MACE, major adverse cardiovascular events

SYNTAX II score	MACE absent, N (%)	MACE present, N (%)	Chi-square value	p-value
Up to 22	264 (78.34%)	73 (21.66%)	218.71	<0.001*
More than 22	60 (19.80%)	243 (80.20%)		

PLR showed no significant correlation with age or gender (p = 0.807 and 0.507, respectively), but it showed strong positive correlations with the U/A ratio, NLR, EF, and SYNTAX I score (Table 4).

**Table 4:** Correlation of PLR with other parameters The asterisk (*) indicates a statistically significant p-value. The data for the correlation of PLR with other parameters were presented using Pearson’s coefficient. A p-value of less than 0.05 was considered statistically significant. EF, ejection fraction; NLR, neutrophil-to-lymphocyte ratio; PLR, platelet-to-lymphocyte ratio; U/A ratio, uric acid-to-albumin ratio

Parameter	R (Pearson coefficient)	p-value
Age	0.01	0.807
U/A ratio	0.81	<0.001*
NLR	0.82	<0.001*
EF	-0.85	<0.001*
SYNTAX I score	0.87	<0.001*

PLR was also higher in patients with a high SYNTAX II score (22 and above; p < 0.001) (Table 5).

**Table 5:** Distribution of PLR according to SYNTAX II score The asterisk (*) indicates a statistically significant p-value. The data were presented as mean ± SD. The unpaired t-test was used to assess statistical significance, and a p-value of less than 0.05 was considered statistically significant. PLR, platelet-to-lymphocyte ratio

Parameter	SYNTAX II ≤ 22 (mean ± SD)	SYNTAX II > 22 (mean ± SD)	t-score	p-value
PLR	299.57 ± 178.68	757.57 ± 198.35	-30.73	<0.001*

The proportion of patients experiencing MACE was higher in NSTEMI and STEMI cases compared with UA cases (p < 0.01) (Table 6).

**Table 6:** Distribution of MACE according to indication The asterisk (*) indicates a statistically significant p-value. The data were presented as numbers and percentages. The chi-square test was used to assess statistical significance, and a p-value of less than 0.05 was considered statistically significant. MACE, major adverse cardiovascular events; NSTEMI, non-ST-elevation myocardial infarction; STEMI, ST-elevation myocardial infarction; UA, unstable angina

Parameter	MACE absent		MACE present		Chi-square value	p-value
	N	%	N	%		
NSTEMI	95	43.38%	124	56.62%	20.80	<0.001*
STEMI	93	45.15%	113	54.85%		
UA	136	63.26%	79	36.74%		

PLR values were significantly higher in patients with MACE compared with patients without MACE across all subgroups (p < 0.001) (Table 7).

**Table 7:** Distribution of PLR according to MACE in the subgroups The asterisk (*) indicates a statistically significant p-value. The data were presented as mean ± SD. The unpaired t-test was used to assess statistical significance, and a p-value of less than 0.05 was considered statistically significant. MACE, major adverse cardiovascular events; NSTEMI, non-ST-elevation myocardial infarction; PLR, platelet-to-lymphocyte ratio; STEMI, ST-elevation myocardial infarction; UA, unstable angina

Subgroup	MACE absent (mean ± SD)	MACE present (mean ± SD)	t-score	p-value
PLR in NSTEMI	266.43 ± 126.60	796.60 ± 147.60	-28.00	<0.001*
PLR in STEMI	254.64 ± 125.10	785.74 ± 139.01	-28.54	<0.001*
PLR in UA	252.36 ± 125.61	754.70 ± 158.02	-24.17	<0.001*

PLR values were significantly higher in patients with high SYNTAX II scores compared with patients with low scores across all subgroups (p < 0.001) (Table 8).

**Table 8:** Distribution of PLR according to SYNTAX II score in the subgroups The asterisk (*) indicates a statistically significant p-value. The data were presented as mean ± SD. The unpaired t-test was used to assess statistical significance, and a p-value of less than 0.05 was considered statistically significant. NSTEMI, non-ST-elevation myocardial infarction; PLR, platelet-to-lymphocyte ratio; STEMI, ST-elevation myocardial infarction; UA, unstable angina

Subgroup	SYNTAX II (up to 22) (mean ± SD)	SYNTAX II (more than 22) (mean ± SD)	t-score	p-value
PLR in NSTEMI	312.93 ± 182.33	775.90 ± 192.72	-18.13	<0.001*
PLR in STEMI	311.32 ± 189.33	767.33 ± 188.85	-17.3	<0.001*
PLR in UA	281.48 ± 167.51	715.57 ± 215.52	-15.28	<0.001*

Univariate regression analysis demonstrated that neutrophil count, platelet count, and lymphocyte count were significantly associated with PLR. However, after adjustment for potential confounding variables in the multivariate regression model, these associations were not statistically significant (Table 9).

**Table 9:** Linear regression analysis for variables associated with PLR The data were presented as β coefficients with 95% CIs obtained from linear regression analysis. Univariate and multivariate linear regression analyses were performed to evaluate the association between clinical and laboratory variables and PLR. Results are presented as regression coefficients (β) with 95% CIs. Variables significant in univariate analysis were included in the multivariate model to adjust for potential confounders. A p-value of less than 0.05 was considered statistically significant. β, regression coefficient; PLR, platelet-to-lymphocyte ratio; U/A ratio, uric acid-to-albumin ratio

Variable	Unadjusted β coefficient (95% CI)	p-value	Adjusted β coefficient (95% CI)	p-value
Age	4.67 (-2.761, 12.013)	0.218	-	-
SYNTAX I	3.65 (-4.230, 11.548)	0.363		
Total leukocyte count	-0.01 (-0.034, 0.012)	0.359		
Neutrophil count	-0.03 (-0.062, -0.011)	0.005	1.646 (-1.31, 4.60)	0.273
Serum creatinine	-27.76 (-224.18, 168.66)	0.781	-	-
Uric acid	-12.42 (-63.38, 38.53)	0.632		
Albumin	-33.95 (-129.53, 61.63)	0.486		
U/A ratio	-2.906 (-105.06, 99.25)	0.955		
Platelet count	0.001 (0.000, 0.002)	0.024	11.295 (-0.40, 22.99)	0.058
Gender	124.78 (-51.21, 300.78)	0.164	-	-
Lymphocyte count	-0.102 (-0.157, -0.046)	<0.001	-26.746 (-57.52, 4.02)	0.088

Regression analysis was also performed to evaluate the association between clinical and laboratory variables and the occurrence of MACE. In the unadjusted analysis, age, SYNTAX I score, SYNTAX II score, albumin, U/A ratio, NLR, hypertension, diabetes mellitus, previous ischemic heart disease, and EF showed significant associations with MACE (Table 10).

**Table 10:** Logistic regression analysis for predictors of MACE The data were presented as regression coefficients (β) with 95% CIs obtained from univariate logistic regression analysis. Univariate logistic regression analysis was performed to evaluate the association between clinical and laboratory variables and the occurrence of MACE. Results are presented as regression coefficients (β) with 95% CIs. A p-value of less than 0.05 was considered statistically significant. β, regression coefficient; DM, diabetes mellitus; EF, ejection fraction; HTN, hypertension; IHD, ischemic heart disease; MACE, major adverse cardiovascular events; NLR, neutrophil-to-lymphocyte ratio; PLR, platelet-to-lymphocyte ratio; U/A ratio, uric acid-to-albumin ratio

Variable	Unadjusted OR (95% CI)	p-value	Adjusted OR (95% CI)	p-value
Age	0.895 (0.877, 0.912)	<0.001	1.020 (0.964, 1.079)	0.472
Gender	0.953 (0.695, 1.306)	0.764	-	-
SYNTAX I	0.964 (0.950, 0.979)	<0.001	0.988 (0.967, 1.010)	0.281
SYNTAX II	0.870 (0.850, 0.890)	<0.001	0.849 (0.792, 0.911)	<0.001
Total leukocyte count	-0.01 (-0.034, 0.012)	0.359	-	-
Neutrophil count	1.000 (1.000, 1.000)	0.049	1.000 (1.000, 1.000)	0.687
Albumin	2.184 (1.786, 2.670)	<0.001	0.853 (0.604, 1.206)	0.369
U/A ratio	0.437 (0.348, 0.548)	<0.001	0.989 (0.663, 1.475)	0.958
Platelet count	1.000 (1.000, 1.000)	0.636	-	-
Lymphocyte count	1.000 (1.000, 1.000)	0.069	-	-
PLR	1.000 (1.000, 1.000)	0.796	-	-
NLR	0.895 (0.844, 0.949)	<0.001	0.957 (0.875, 1.047)	0.338
HTN	0.567 (0.411, 0.782)	0.001	0.890 (0.528, 1.499)	0.661
DM	0.531 (0.322, 0.876)	0.013	0.405 (0.215, 0.763)	0.005
Old IHD	1.728 (1.112, 2.686)	0.015	1.917 (1.026, 3.580)	0.041
EF	1.103 (1.079, 1.128)	<0.001	1.047 (1.012, 1.083)	0.008
Indication	1.364 (0.977, 1.903)	0.068	-	-

A Mann-Whitney U test was performed to compare continuous clinical and laboratory parameters between patients with and without MACE, as several variables did not satisfy normality assumptions. Significant differences were observed for age, SYNTAX I score, SYNTAX II score, neutrophil count, serum creatinine, uric acid, albumin, U/A ratio, lymphocyte count, PLR, NLR, and EF (Table 11).

**Table 11:** Mann-Whitney U test comparing clinical and laboratory parameters between patients with and without MACE The data were presented as Mann-Whitney U statistic, Wilcoxon W statistic, standardized Z value, and two-tailed p-values obtained from nonparametric comparisons between the two groups (MACE present vs MACE absent). The Mann-Whitney U test was used to compare continuous variables because several variables did not follow a normal distribution. A p-value of less than 0.05 was considered statistically significant. EF, ejection fraction; MACE, major adverse cardiovascular events; NLR, neutrophil-to-lymphocyte ratio; PLR, platelet-to-lymphocyte ratio; U/A ratio, uric acid-to-albumin ratio

Variable	Mann-Whitney U statistic	Z	p-value
Age	20,868.0	-12.972	0.0
SYNTAX I	39,125.5	-5.2	0.0
SYNTAX II	18,362.0	-14.039	0.0
Total leukocyte count	48,268.0	-1.186	0.236
Neutrophils (%)	2,641.5	-0.722	0.47
Neutrophil count	46,429.0	-2.037	0.042
Serum creatinine	37,170.5	-5.996	0.0
Uric acid	43,071.5	-3.473	0.001
Albumin	32,695.5	-7.915	0.0
U/A ratio	33,542.5	-7.547	0.0
Platelet count	49,833.5	-0.515	0.607
Lymphocyte count	43,969.0	-3.089	0.002
PLR	45,034.0	-2.572	0.01
NLR	42,484.0	-3.665	0.0
EF	29,800.0	-9.346	0.0

Correlation analysis was performed to evaluate the relationship between SYNTAX II score and various clinical and laboratory parameters. Significant positive correlations were observed between SYNTAX II score and SYNTAX I score, serum creatinine, uric acid, U/A ratio, PLR, and NLR. Significant negative correlations were observed with albumin, lymphocyte count, and EF (Table 12).

**Table 12:** Correlation analysis between SYNTAX II score and clinical and laboratory parameters The data were presented as Spearman correlation coefficients (r) with corresponding p-values. Spearman correlation analysis was performed to evaluate the relationship between the SYNTAX II score and selected clinical and laboratory variables. The table presents the correlation coefficient (r) and corresponding p-value for each variable. EF, ejection fraction; NLR, neutrophil-to-lymphocyte ratio; PLR, platelet-to-lymphocyte ratio; U/A ratio, uric acid-to-albumin ratio

Parameter	Correlation coefficient	p-value
SYNTAX I	0.205	<0.001
Total leukocyte count	-0.001	0.983
Neutrophil count	0.044	0.262
Serum creatinine	0.366	<0.001
Uric acid	0.289	<0.001
Albumin	-0.651	<0.001
U/A ratio	0.611	<0.001
Platelet count	-0.074	0.061
Lymphocyte count	-0.222	<0.001
PLR	0.148	<0.001
NLR	0.185	<0.001
EF	-0.511	<0.001

Pearson correlation analysis was performed to evaluate the relationship between PLR and selected clinical and laboratory parameters. PLR showed significant positive correlations with SYNTAX I score, U/A ratio, NLR, and duration of hospital stay, while a significant negative correlation was observed with EF. No significant correlation was observed between PLR and age (Table 13).

**Table 13:** Pearson correlation analysis between PLR and selected clinical and laboratory parameters The data were presented as Pearson correlation coefficients (r), corresponding two-tailed p-values, and the number of observations (N). Pearson correlation analysis was performed to evaluate the relationship between PLR and the selected variables. A p-value of less than 0.05 was considered statistically significant. EF, ejection fraction; NLR, neutrophil-to-lymphocyte ratio; PLR, platelet-to-lymphocyte ratio; U/A ratio, uric acid-to-albumin ratio

Parameter	PLR	Age	SYNTAX I	U/A ratio	NLR	EF	Duration
PLR	Pearson Correlation	1	0.01	0.87	0.84	0.82	-0.85
	Significance (two tailed)	-	0.807	0	0	0	0
	N	640	640	640	640	640	640
Age	Pearson Correlation	0.01	1	0	0	-0.01	0.01
	Significance (two tailed)	0.807		0.963	0.903	0.733	0.723
	N	640	640	640	640	640	640

**Figure 1:**
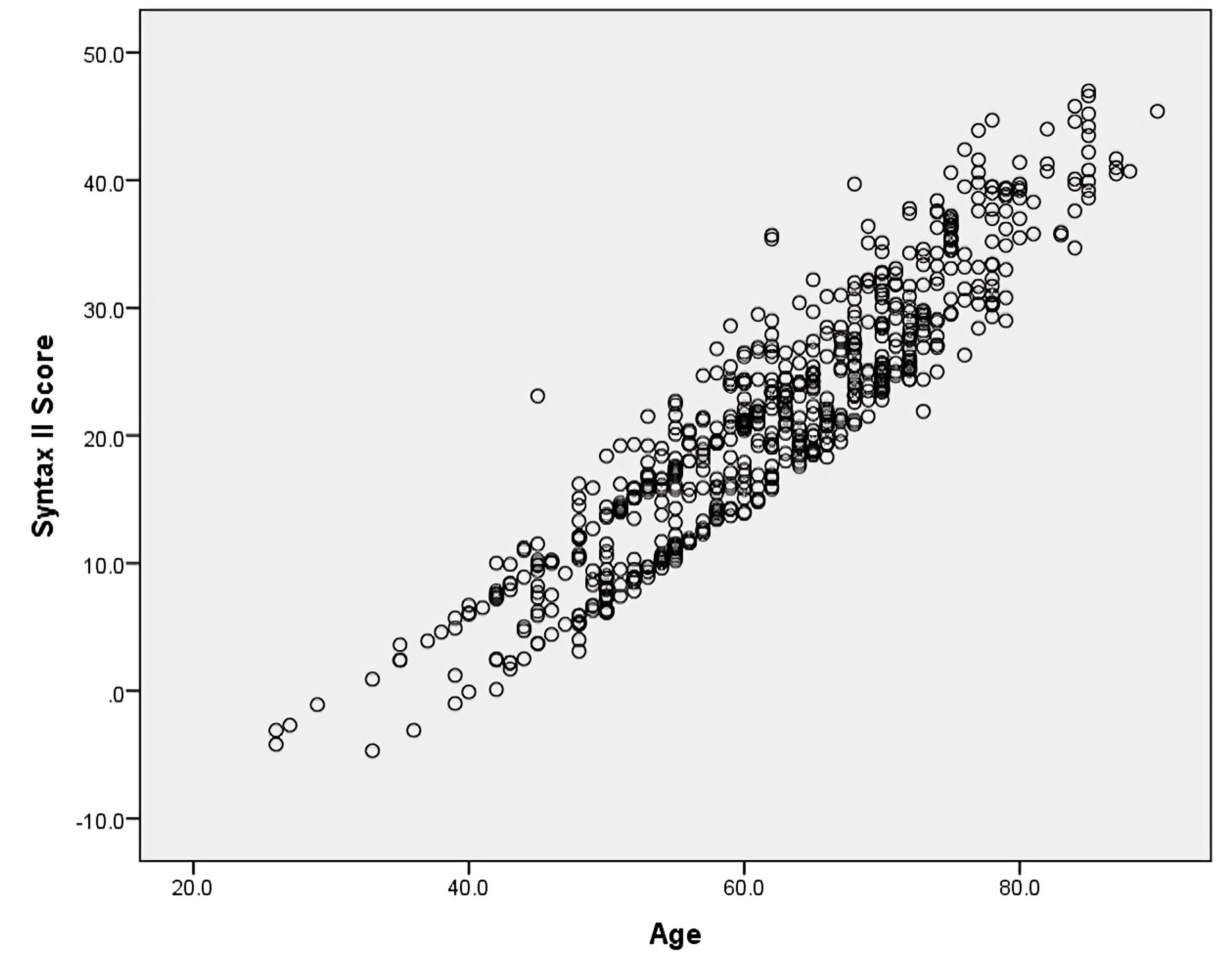
Scatter plot showing correlation between age and SYNTAX II score Each point represents an individual patient in the study population. Age (years) is shown on the x-axis and SYNTAX II score on the y-axis. Pearson correlation analysis demonstrated a statistically significant positive association between age and SYNTAX II score (r = 0.62, p < 0.001), suggesting increasing coronary artery disease complexity with increasing age.

Scatter plot analysis demonstrated a significant positive correlation between age and SYNTAX II score, indicating that angiographic complexity increased with advancing age (r = 0.62, p < 0.001) (Figure 1).

**Figure 2:**
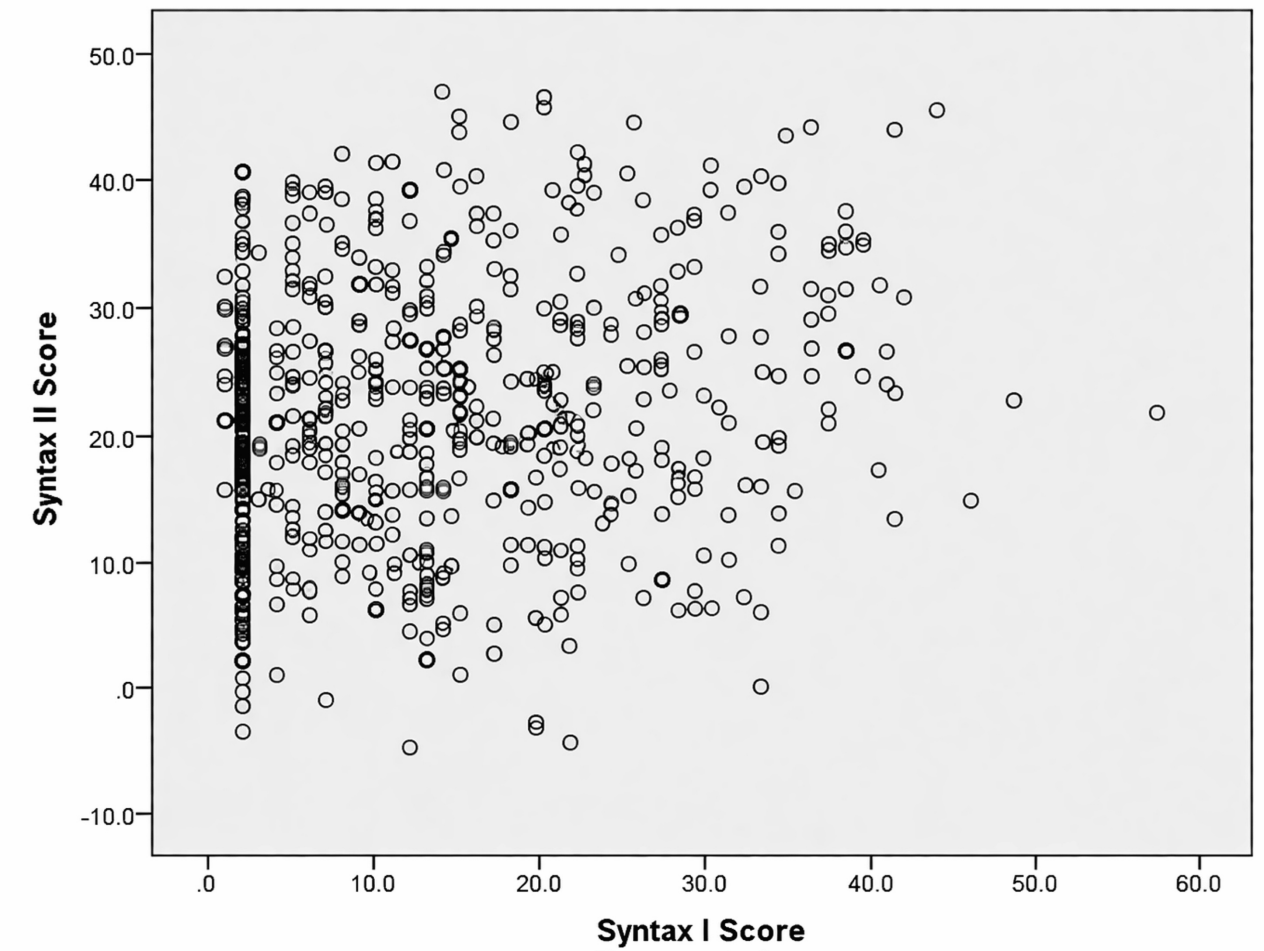
Scatter plot showing correlation between SYNTAX I score and SYNTAX II score Each point represents a patient included in the analysis. SYNTAX I score is plotted on the x-axis and SYNTAX II score on the y-axis. Pearson correlation analysis demonstrated a weak positive relationship between the two scores (r = 0.205, p < 0.001), indicating partial agreement between anatomical and clinical risk assessment systems.

A weak but statistically significant positive association was observed between SYNTAX I score and SYNTAX II score, indicating partial concordance between the two angiographic scoring systems (r = 0.205, p < 0.001) (Figure 2).

**Figure 3:**
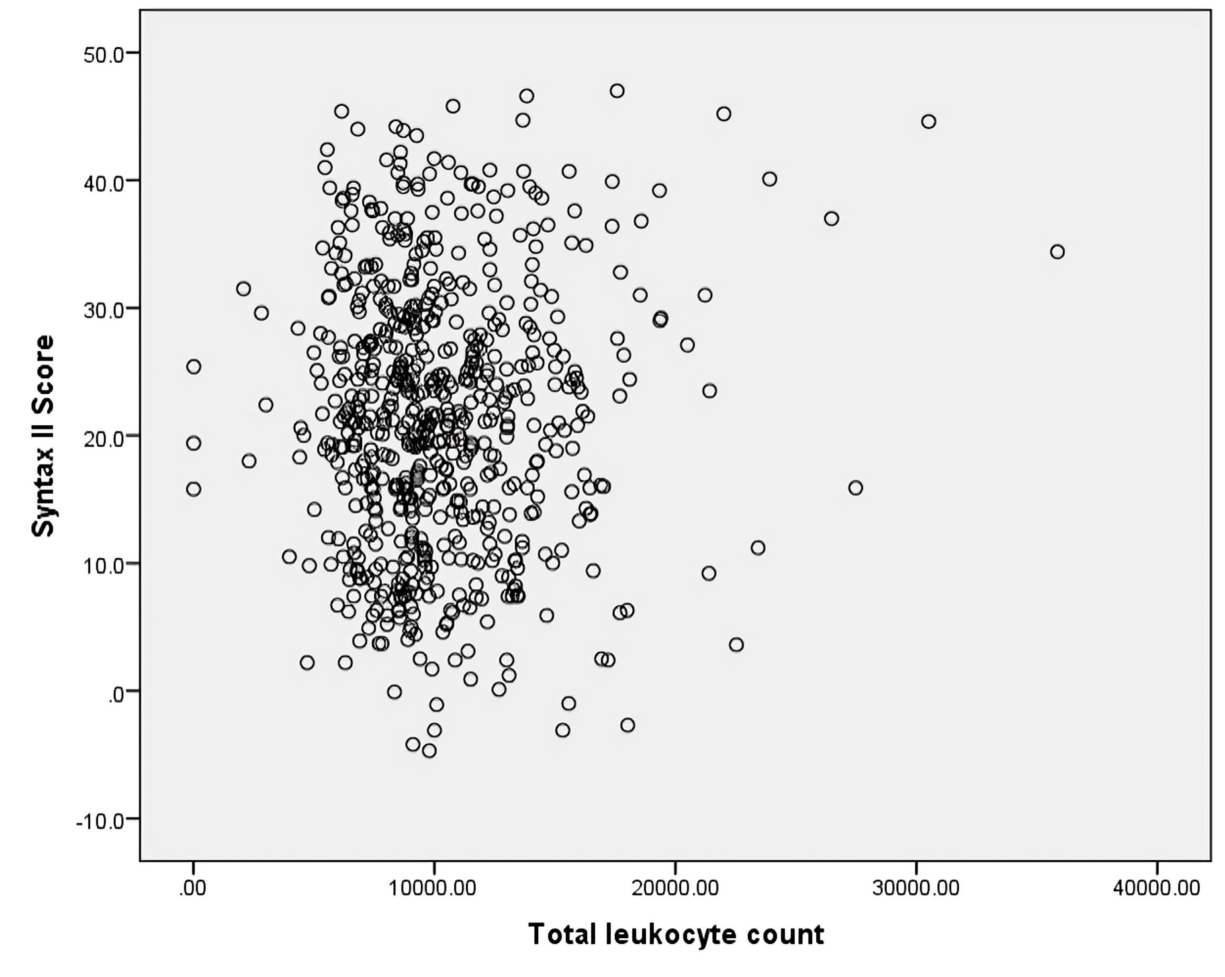
Scatter plot showing correlation between total leukocyte count and SYNTAX II score Each point represents an individual patient. Total leukocyte count is displayed on the x-axis and SYNTAX II score on the y-axis. Pearson correlation analysis demonstrated no significant relationship between total leukocyte count and angiographic severity (r = -0.001, p = 0.983).

No statistically significant correlation was observed between total leukocyte count and SYNTAX II score, suggesting limited association between leukocytosis and angiographic disease severity (r = -0.001, p = 0.983) (Figure 3).

**Figure 4:**
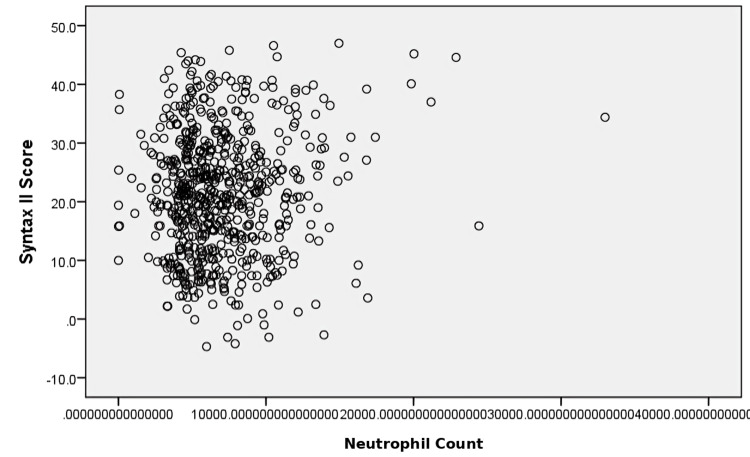
Scatter plot showing correlation between neutrophil count and SYNTAX II score Each point represents an individual patient. Neutrophil count is plotted on the x-axis and SYNTAX II score on the y-axis. Pearson correlation analysis demonstrated a weak but statistically nonsignificant association between neutrophil count and SYNTAX II score (r = 0.044, p = 0.262).

A weak positive association was observed between neutrophil count and SYNTAX II score; however, this relationship was not statistically significant (r = 0.044, p = 0.262) (Figure 4).

**Figure 5:**
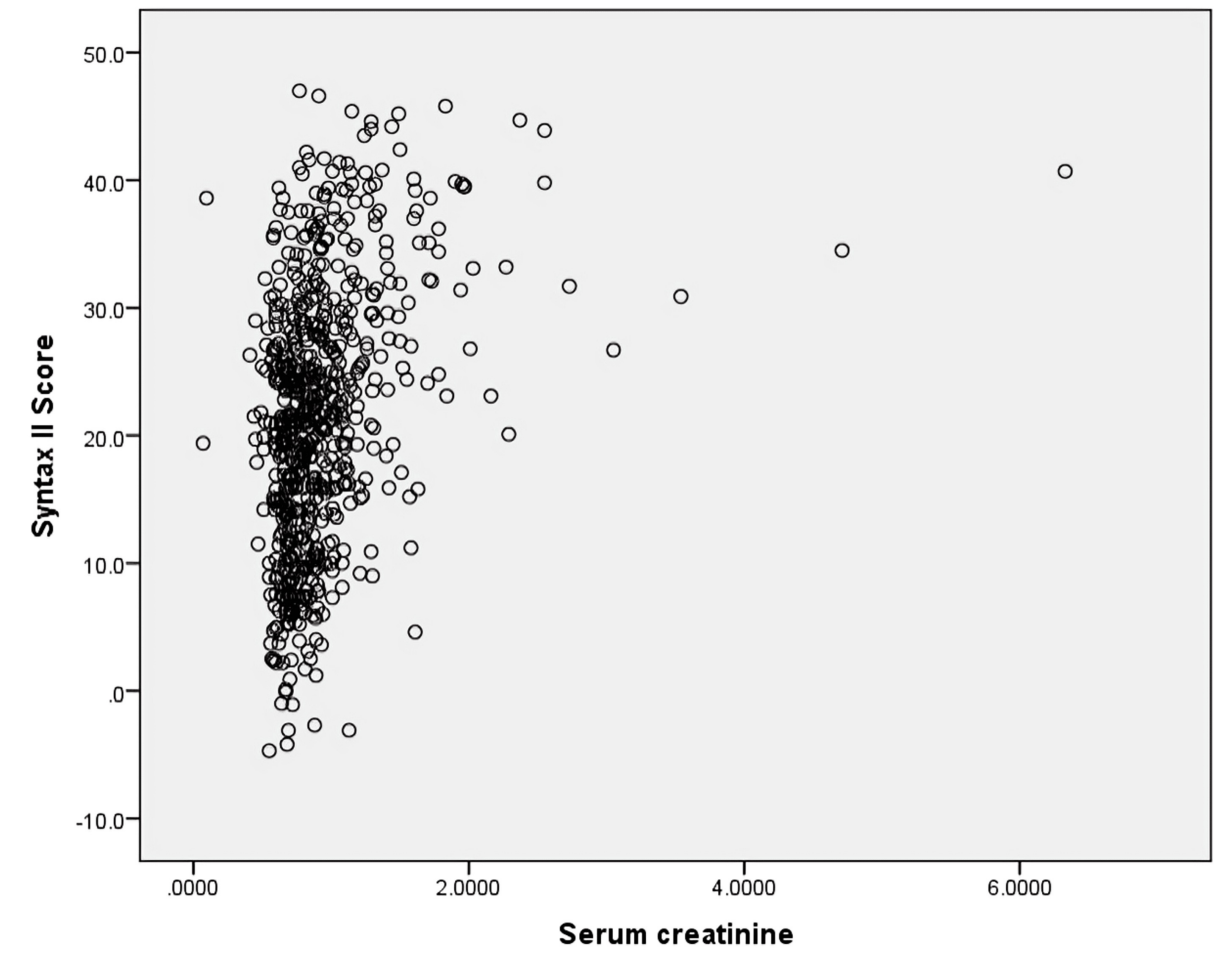
Scatter plot showing correlation between serum creatinine and SYNTAX II score Each point represents a patient in the study population. Serum creatinine (mg/dL) is shown on the x-axis and SYNTAX II score on the y-axis. Pearson correlation analysis demonstrated a statistically significant moderate positive association between serum creatinine and SYNTAX II score (r = 0.366, p < 0.001).

A moderate positive correlation was observed between serum creatinine and SYNTAX II score, indicating that higher creatinine levels were associated with greater angiographic disease complexity (r = 0.366, p < 0.001) (Figure 5).

**Figure 6:**
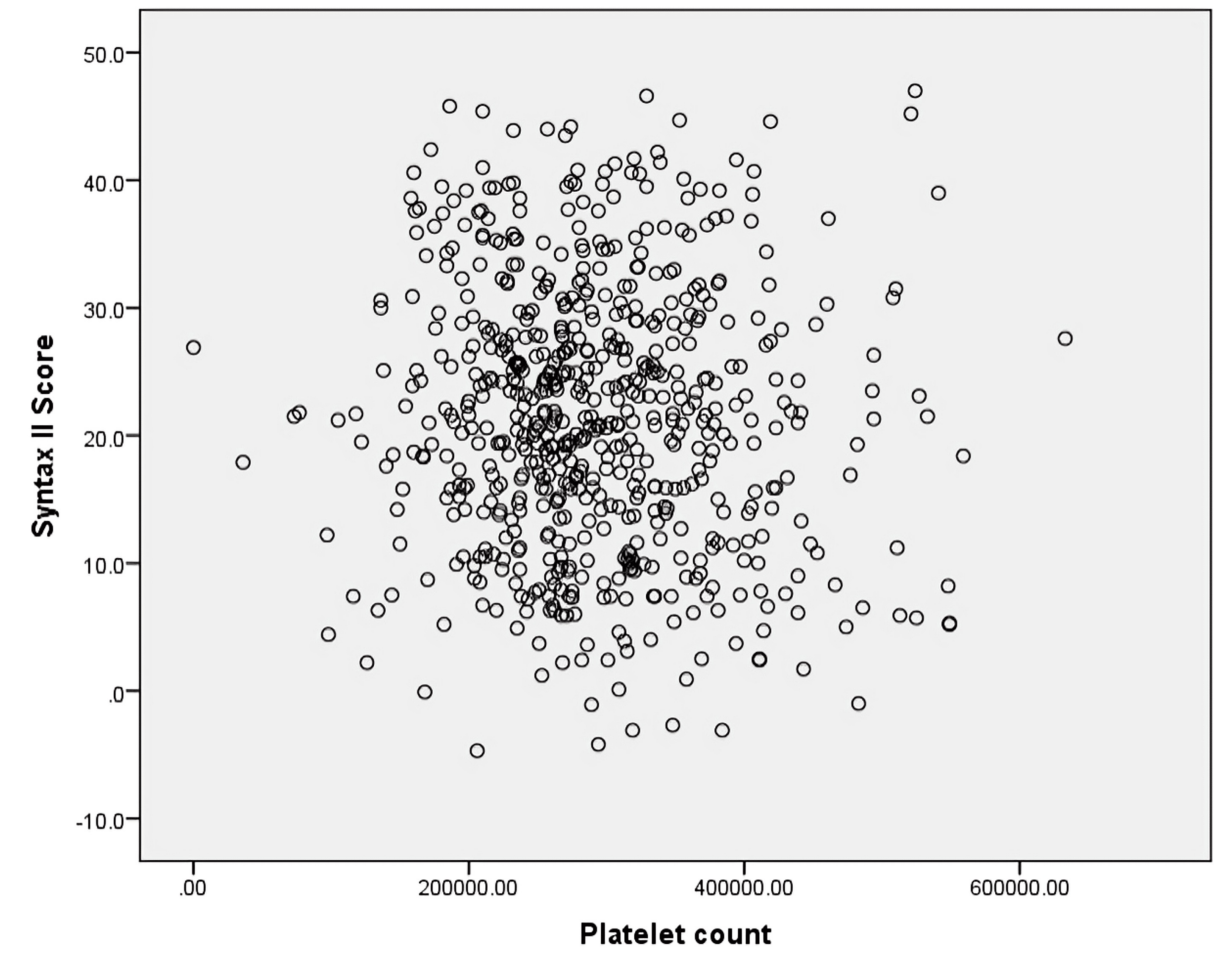
Scatter plot showing correlation between platelet count and SYNTAX II score Each point represents an individual patient. Platelet count is displayed on the x-axis and SYNTAX II score on the y-axis. Pearson correlation analysis demonstrated no statistically significant relationship between platelet count and angiographic severity (r = -0.074, p = 0.061).

Scatter plot analysis showed no statistically significant correlation between platelet count and SYNTAX II score (r = -0.074, p = 0.061) (Figure 6).

## Discussion

In the present study, PLR correlated strongly with the SYNTAX scores and other indicators of inflammation, such as NLR. Patients with MACE also had significantly higher PLR than patients without MACE. From a clinical perspective, PLR may provide early risk enrichment at the time of presentation using routinely available laboratory data. Elevated PLR could help identify ACS patients at higher risk for adverse in-hospital events who may benefit from closer hemodynamic monitoring, early cardiology involvement, or prompt invasive evaluation. Importantly, PLR should be viewed as an adjunct to established clinical assessment and angiographic scoring systems rather than as a replacement.

Over the years, there has been a search for readily available indicators to predict MACE in ACS patients. Traditional scoring systems, such as the Global Registry of Acute Coronary Events (GRACE) score, can be cumbersome and somewhat outdated. PLR is one such indicator. In the meta-analysis by Li et al., 10 studies including 8,932 ACS patients were analyzed. They reported that the risk of in-hospital MACE was 2.24-fold higher in the high PLR group, and the risk of long-term adverse outcomes was 2.32-fold higher [10]. Another meta-analysis predicted the risks to be 1.95-fold and 1.50-fold, respectively [11]. In the present study, the MACE group had higher PLR, which is comparable to these findings.

Further studies have assessed the prognostic efficiency of PLR in patient subgroups. Ugur et al. conducted a study on STEMI patients undergoing percutaneous coronary intervention (PCI) and reported that PLR was significantly associated with six-month all-cause mortality, with 7% deaths in the high PLR group compared to 3% in the low PLR group (p = 0.03) [12]. Temiz et al. included 636 STEMI patients and reported similar results, with high PLR associated with higher in-hospital mortality. They also found that a PLR greater than 144 was an independent predictor of in-hospital mortality [13].

Li et al. studied 429 NSTEMI patients undergoing CAG and observed that patients with MACE had higher PLR (p = 0.007), higher NLR (p < 0.001), and higher SYNTAX II scores (p = 0.008). They reported that all these parameters are independent risk factors for coronary artery disease severity and that SYNTAX score was associated with high NLR and PLR [14]. Similar results were reported by Bećirović et al. [15]. Thus, the prognostic efficiency of PLR in different patient subsets has been established.

Other studies have assessed the correlation of PLR and NLR with the SYNTAX score, an established predictor of MACE. Kurtul et al. conducted a study on 1,016 patients undergoing CAG and found that high SYNTAX scores were associated with high PLR. They also reported that a PLR of 116 or above was a significant predictor of intermediate-to-high SYNTAX score, with 71% sensitivity and 66% specificity [16]. Similar results were reported by Karadeniz et al. [17], who found that NLR had a significantly positive correlation with SYNTAX score (r = 0.58, p < 0.001) and that patients with high PLR also had high SYNTAX scores (p < 0.001). These findings are comparable to the present study, where strong, significant positive correlations were observed between SYNTAX score, PLR, and NLR. The studies cited in this section were selected narratively to contextualize our findings and were not derived from a systematic review. Differences across published studies, including variation in ACS subtype distribution, timing of blood sampling relative to symptom onset and initiation of therapies, exclusion of noncardiac inflammatory conditions, and heterogeneity in outcome definitions and follow-up duration, may contribute to differences in the strength of association between PLR and clinical outcomes. Direct numerical comparison of effect sizes across studies is limited by heterogeneity in PLR cutoffs, timing of measurement, and differences in endpoints; nevertheless, the direction of association in our cohort is consistent with the broader literature, supporting PLR as a risk-enrichment marker in ACS.

Studies have also explored correlations between PLR and traditional predictors of adverse outcomes. Zhou et al. investigated the relationship of PLR with GRACE score and observed that, after a 58-month follow-up, both indicators independently predicted adverse events. They recommended that combining these two indicators significantly improves predictability for adverse outcomes [18]. PLR and NLR were also significantly associated with GRACE score in STEMI patients [19]. Kurtul et al. showed that PLR was strongly associated with thrombolysis in myocardial infarction (TIMI) flow grade after primary PCI, with high PLR predicting low TIMI grades [20]. Collectively, these studies establish PLR as a prognostic indicator of MACE.

PLR reflects ongoing systemic inflammation, integrating two key parameters: high platelets and low lymphocytes. The pathophysiological mechanisms linking elevated PLR to poor prognosis in ACS are multifactorial. Elevated platelet levels act as both an outcome and a trigger for the inflammatory response. Inflammatory mediators such as interleukin-6, CRP, and tumor necrosis factor-α stimulate platelet proliferation [21]. Platelets also release thromboxane A₂, promoting monocyte adhesion and migration, which leads to inflammation and plaque weakening [22,23]. The procoagulant function of platelets in homeostasis and thrombosis may also contribute to arterial thrombus progression [24]. This thrombotic process and plaque weakening are closely associated with MACE. Lymphocytes are important in myocardial healing [25], and low levels can delay recovery, potentially leading to complications. Lymphopenia may result from circulating cortisol in response to stress. Thus, high PLR is a potential predictor of MACE in ACS patients.

Although PLR reflects thrombo-inflammatory activation relevant to ACS, it is a nonspecific biomarker and may also be elevated in noncardiac conditions such as infections, malignancies, or other inflammatory diseases. This underscores the importance of interpreting PLR within the appropriate clinical context and highlights the value of stringent exclusion criteria in minimizing confounding when evaluating its prognostic significance in ACS. PLR can also be influenced by comorbid conditions and concurrent therapies. Although exclusion criteria were applied to reduce major noncardiac inflammatory confounding, residual confounding may remain, including medication exposure, baseline comorbidities, and treatment variability during hospitalization. Standard ACS management includes antiplatelet therapy, anticoagulation when indicated, statins, and guideline-directed secondary prevention. The optimal duration of some therapies continues to evolve. For example, recent meta-analyses suggest that beta-blockers may not confer additional mortality benefit beyond a one-year event-free period after myocardial infarction in patients without reduced EF, emphasizing the need to individualize therapy [26]. Our study did not evaluate treatment effects or long-term medication outcomes, which were outside the scope of this analysis.

Social determinants of health are increasingly recognized as independent predictors of cardiovascular outcomes. Although not measured in our cohort, future work should evaluate whether integrating PLR with established risk scores and social determinants improves risk stratification [27].

Limitations

There are several limitations to this study that should be considered when interpreting the findings. First, although significant associations between PLR and in-hospital MACE were observed, the absence of multivariate adjustment for potential confounders precludes confirmation of PLR as an independent predictor of adverse outcomes. Therefore, these findings should be interpreted as associative rather than causal.

Second, as a prospective observational study, causal relationships cannot be inferred. Follow-up was limited to hospitalization, and the prognostic value of PLR for long-term outcomes such as post-discharge mortality, reinfarction, or rehospitalization was not assessed. Third, the single-center nature of the study may limit generalizability to other populations with different demographics, comorbidity burdens, or treatment practices. The observed female preponderance in our cohort may reflect regional referral patterns and could influence external applicability.

Fourth, PLR is a nonspecific biomarker derived from routine hematologic parameters and may be influenced by physiological stress, inflammatory states, or laboratory variability. Measurement at a single time point may not capture dynamic changes in inflammatory status during ACS. Finally, while the overall sample size was adequate for primary analyses, the relatively short study duration and subgroup sizes may have limited statistical power to detect smaller differences or interactions across ACS subtypes.

These limitations highlight the need for larger, multicenter studies with longer follow-up and comprehensive adjustment for confounders to further clarify the independent prognostic role of PLR in ACS.

## Conclusions

In this prospective observational study, higher PLR was associated with greater angiographic disease severity and a higher incidence of in-hospital MACE among patients presenting with ACS. PLR also demonstrated significant correlations with established indicators of disease severity, including SYNTAX scores and NLR. However, given the observational design, these findings demonstrate association rather than causation, and PLR cannot be considered an independent causal predictor of adverse outcomes. Although prior studies have suggested various PLR cutoff values for risk stratification, the optimal threshold remains inconsistent across populations, and the present study was not designed to establish a definitive cutoff for clinical use.

PLR is a simple and readily available biomarker that may serve as an adjunctive indicator of inflammatory and thrombotic activity in ACS. It should be interpreted alongside established clinical and angiographic risk assessment tools rather than as a substitute. This study provides prospective evidence from an Indian cohort encompassing all major ACS subtypes and uniquely integrates PLR with angiographic disease severity and in-hospital clinical outcomes. Further large, multicenter prospective studies with longer follow-up and mechanistic evaluation are required to validate these findings and clarify the role of PLR in risk stratification of ACS patients.
